# US Consumer Identification of the Health Benefits of Dietary Fiber and Consideration of Fiber When Making Food Choices

**DOI:** 10.3390/nu14112341

**Published:** 2022-06-03

**Authors:** Christopher R. Gustafson, Devin J. Rose

**Affiliations:** 1 Department of Agricultural Economics, University of Nebraska-Lincoln, Lincoln, NE 68583, USA; 2 Department of Food Science and Technology, University of Nebraska-Lincoln, Lincoln, NE 68588, USA; 3 Department of Agronomy & Horticulture, University of Nebraska-Lincoln, Lincoln, NE 68583, USA; 4 Nebraska Food for Health Center, University of Nebraska-Lincoln, Lincoln, NE 68588, USA

**Keywords:** nutritional knowledge, food labeling, under-consumed nutrients, consumer choice

## Abstract

The purposes of this study were to (1) determine beliefs in the health benefits of dietary fiber, an under-consumed nutrient of public health concern, and (2) determine the relationship between beliefs about dietary fiber and consideration of fiber when making food choices. We conducted a nationally representative within-subject randomized online survey of 42,018 US primary shoppers in May–June 2021. Participants selected health benefits they believed were associated with consumption of fiber from a list of six benefits recognized by the Food and Drug Administration (FDA), one indirect benefit, and one unrelated benefit. Respondents then indicated which nutrients, if any, they considered when making food choices. Respondents selected 1.77 (95% CI = 1.76–1.77) FDA-recognized benefits out of a total six; half (50.6%) of respondents identified zero or one FDA-recognized benefit. The most-cited benefit was “improving bowel movements” (64.4%). Older participants perceived significantly more FDA-recognized fiber benefits. Identification of FDA-recognized benefits increased odds ratios for consideration of fiber during food choice (relative to zero benefits) from 3.0 for one benefit (95% CI = 2.8–3.3) to 14.3 for six benefits (95% CI = 12.4–16.6). Consumers are largely unaware of the many health benefits of dietary fiber, which dramatically decreases the likelihood that they consider this important, under-consumed nutrient during food choice.

## 1. Introduction

Low quality diet, which contributes to heart disease, certain cancers, and type-2 diabetes, is the leading risk factor for premature morbidity and mortality, and chronic disability, in the United States (US) [1,2]. The health care costs of treating diet-related chronic diseases exceed $50 billion per year [3]. While the need for strategies to improve diet quality of the public has been recognized for decades, the main national policy approaches, which have focused on providing objective nutrition information about dietary components in retail settings, have had limited success [4,5,6].

The Dietary Guidelines for Americans (DGA), a document compiled by the US Department of Agriculture and the Department of Health and Human Services, provides guidance for healthy dietary patterns [7]. In addition to describing the nutrients that people should limit consumption of—such as sodium, saturated fat, and added sugar—the DGA also highlights nutrients that are of public health concern because they are under-consumed by many Americans. These nutrients are dietary fiber, calcium, potassium, and vitamin D. Strategies to increase consumption of foods high in these nutrients would improve the diet quality and overall health of the public.

Among these nutrients, dietary fiber may be particularly valuable for reducing many of the diet-related diseases that plague modern society. Indeed, dietary fiber is associated with reduced risk of coronary heart disease and type-2 diabetes, which ranked first and eighth, respectively, among all causes of mortality in the United States in 2019 and 2020 [1,8,9].

Since their inception, the US Dietary Guidelines have emphasized the importance of a high fiber diet. Yet, after more than four decades, less than 3% of men and 6% of women consume the recommended 14 g dietary fiber/1000 kcal [10,11,12,13]. Based on the 2015–2018 NHANES dietary survey, median dietary fiber intake for men and women was a mere 7.3 g/1000 kcal and 8.3 g/1000 kcal, respectively [13].

A recent qualitative study on dietary fiber in Australia showed that while many people understood that experts recommend consuming high-fiber foods, they reported being largely unaware of the health benefits of dietary fiber consumption [14]. This is of concern because nutritional knowledge has been shown to be an important driver of consumption of healthy food groups—such as fruits and vegetables [15]. This likely also applies to consumption of important nutrients such as dietary fiber; however, the public’s knowledge about recognized health benefits of under-consumed nutrients has not been studied in a large, representative sample of Americans.

The US Food and Drug Administration (FDA) has established a list of recognized health benefits of dietary fiber [11]. These FDA-recognized benefits are lowering blood glucose, lowering cholesterol levels, lowering blood pressure, increasing frequency of bowel movements, increasing mineral absorption in the intestinal tract, and reducing energy intake [11]. One or more of these benefits must be established in any new non-digestible carbohydrate ingredient to be labelled as dietary fiber.

In addition to these FDA-recognized health benefits, recent research has established that fermentation by the microbiome is critical for bringing about many of the health benefits of dietary fibers [16,17]. Accordingly, there has been a recent increase in popular books and websites aimed at improving human gut health [18,19,20,21,22,23,24]. Although the terms “healthy microbiome” or “healthy bacteria” have not been completely defined in the scientific literature, books and website often use these terms presumably to improve consumer understanding. Therefore, promoting healthy gut bacteria may be considered by some consumers as an indirect benefit of dietary fiber even if it is not part of the FDA-recognized list of benefits for regulatory purposes.

We hypothesized that many individuals are unaware of the numerous and diverse health benefits of dietary fiber. Further, given that nutritional knowledge is an important driver of consumption of healthy foods [15], we hypothesized that awareness of (more) health benefits of dietary fiber increases the likelihood that an individual considers dietary fiber content during food choice. Therefore, the purposes of this study were twofold: (1) to determine the distribution of beliefs about the health benefits of dietary fiber in a large, population-weighted US consumer panel; and (2) to determine the relationship between beliefs about the benefits of dietary fiber and consideration of fiber when making food choices.

## 2. Materials and Methods

### 2.1. Survey

In this research, we examined knowledge of FDA-recognized health benefits of fiber and consideration of fiber during food choice among 42,018 US participants in the National Consumer Panel Omnibus Survey (Information Resources, Inc., IRi; Chicago, IL, USA). This is a bi-monthly survey of members of a nationwide consumer panel fielded by IRi, with half of the panel completing the survey each month. In the May–June 2021 survey cycle, we included questions about health benefits of dietary fiber consumption and consideration of fiber during food choice. This study was conducted according to the guidelines outlined in the Declaration of Helsinki, and all procedures involving research study participants were approved by the University of Nebraska–Lincoln’s Institutional Review Board (IRB protocol #20201020721EX). All participants provided electronic informed consent before participating in the research.

### 2.2. Measures

Respondents selected health benefits they believed were associated with consumption of dietary fiber from a list of eight potential benefits, which were presented in a random order (Appendix A). The list included the six benefits recognized by the FDA, as well as two other items: “Supporting healthy gut bacteria,” which is an indirect benefit of dietary fiber; and “Improving skin complexion,” which does not have any documented relationship to dietary fiber intake. The number of benefits participants could indicate that they believe result from dietary fiber consumption ranged from 0 (no benefits selected) to 8 (all benefits selected). The responses were not ranked by participants, but simply selected from the list. Importantly, we phrased the question as a “belief” question (e.g., “Which of the following … do you believe are health benefits that result from consuming dietary fiber…”) rather than a “quiz” question (e.g., “Which of the following benefits is associated with dietary fiber…”), where consumers may be more likely to limit the number of benefits they selected. After the respondent indicated the health benefits they perceived as stemming from consumption of dietary fiber, they indicated the nutrients they consider (or had considered in the past when establishing current dietary patterns) when choosing which foods to buy or eat from a list of eight nutrients. These nutrients included dietary fiber in addition to seven other nutrients that are required to appear on all food labels in the US.

### 2.3. Data Analysis

The responses to the questions about fiber health beliefs were used in combination with respondent-specific population weights, which were provided with the data by the survey firm. We used the responses and weights to calculate weighted means and 95% confidence intervals of participants’ beliefs of the health benefits of dietary fiber, using the “weighted.mean” and “weighted.sd” commands in RStudio.

We conducted a multivariate ordinal logistic regression of the number of FDA-recognized health benefits that participants selected on gender and age to examine associations between demographic characteristics and fiber beliefs. Finally, we conducted a multivariate binary logistic regression of consideration of dietary fiber when making food choices on the number of FDA-recognized fiber health benefits a participant identified and demographic variables to examine the relationship between the number of health benefits selected and the likelihood that an individual incorporates information about dietary fiber into food choice. In the ordinal logistic regression, the variable “number of FDA-recognized health benefits” was treated as an ordered categorical variable. Data were analyzed and visualized using R and R Studio using the “radiant.data,” “nnet”, “MASS”, “ggplot2”, and “cowplot” packages [25,26,27,28,29,30].

## 3. Results

Inasmuch as the purposes of this study were to gather information about consumer beliefs about the health benefits of dietary fiber and to determine associations between these beliefs and food choice, we gathered data from a population-weighted consumer panel of US primary shoppers. The panel consisted of primary household shoppers and thus reflects a primarily female and older demographic (Table 1), which is reflective of US grocery shoppers in general [31,32,33].

**Figure 1:**
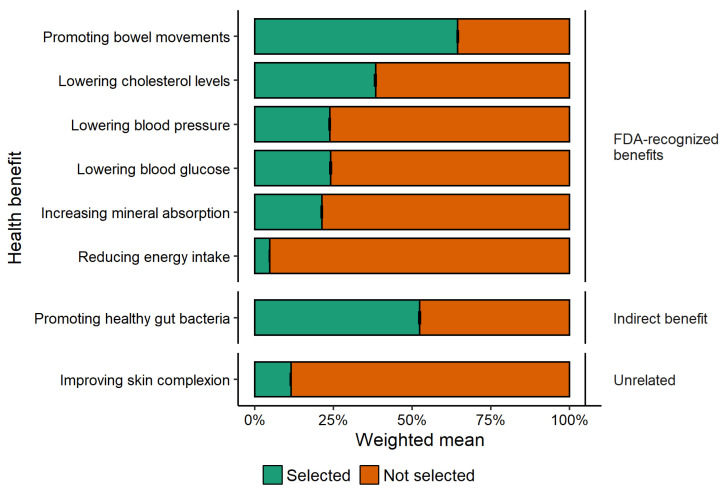
Proportion of respondents that selected or did not select FDA-recognized health benefits of dietary fiber, an indirect benefit, and one unrelated benefit in the IRi survey; error bars show 95% confidence interval (*n* = 42,018); some error bars may be too close to the mean to see.

The most frequently perceived FDA-recognized health benefit of dietary fiber was increased frequency of bowel movements (Figure 1). All other FDA-recognized benefits were identified by <40% of respondents. One FDA-recognized benefit—reduced energy intake—was only perceived by 4.7% of respondents, which was less than the unrelated benefit of improving skin complexion (11.5%). The second most frequently cited benefit of dietary fiber was promoting healthy gut bacteria, which was indicated as a health benefit of dietary fiber by 52.4% of respondents.

**Figure 2:**
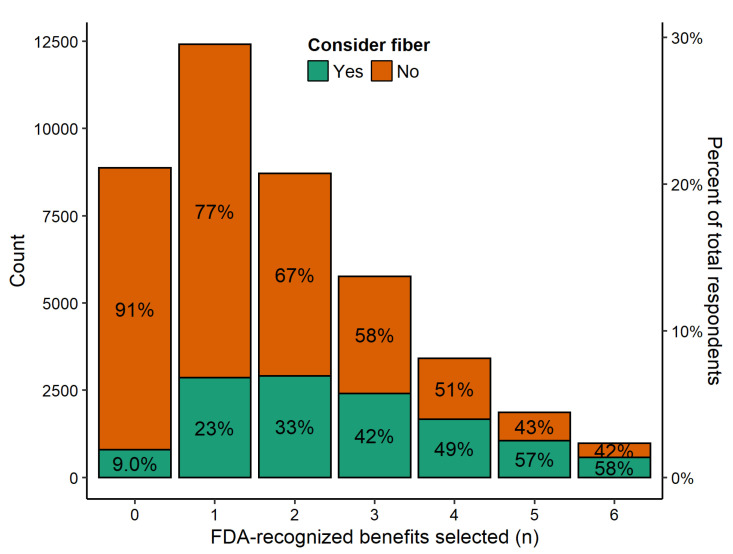
Distribution of the number of FDA-recognized health benefits selected by participants in the IRi survey; bars are colored by whether participants indicated they consider fiber when making food choices; percentages within bars are the proportion of individuals selecting the given number of FDA-recognized benefits that considered or did not consider fiber when making food choices; the percentage of respondents given on the right *y*-axis is the proportion of the entire sample that selected the given number of FDA-recognized benefits.

In the IRi survey, respondents selected only 1.77 (95% CI = 1.76–1.77) FDA-recognized benefits out of a total of six. More than 1 in 5 participants selected none of the FDA-recognized health benefits and over 50% of respondents selected zero or only one recognized benefit (Figure 2). Only 2.3% of respondents correctly perceived all six recognized health benefits. The proportion of individuals that consider fiber when making food choices increased with each additional benefit selected from only 9% of those that identified no benefits of fiber to 58% of those that identified all six FDA-recognized benefits of fiber.

The ordinal logistic regression analysis of FDA-recognized benefits on demographic characteristics showed that age had the largest impact on the number of FDA-recognized dietary fiber benefits selected (Table 2). Relative to the youngest age group (19–24-year-olds), each additional age category increased the number of FDA-recognized fiber benefits identified. Female respondents were also slightly but significantly more likely to perceive higher numbers of fiber benefits compared with respondents who were not female.

**Figure 3:**
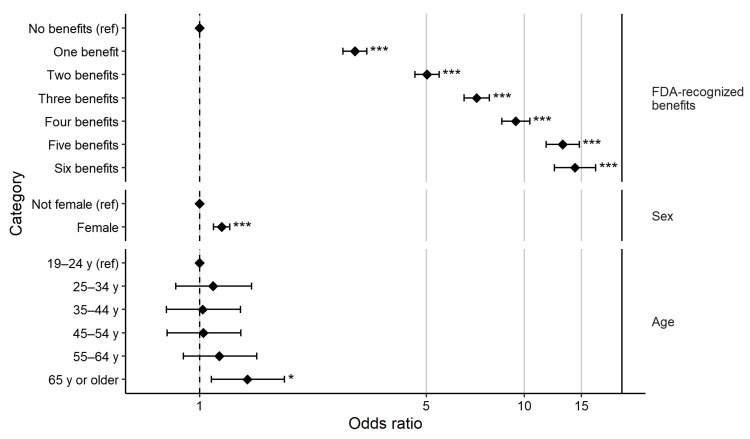
Multivariate binary logistic regression of consideration of dietary fiber when making food choices (versus not considering fiber); error bars show 95% confidence interval; * *p* < 0.05, *** *p* < 0.001.

Next, we examined how beliefs in the health benefits of dietary fiber affected the likelihood that an individual considered dietary fiber content during food choice. Overall, 12,278 respondents (29.2%) indicated that they considered fiber when making food choices. The multivariate binary logistic regression of consideration of dietary fiber during food choice on the number of FDA-recognized fiber health benefits selected and demographic characteristics showed that the number of health benefits a person believed stemmed from dietary fiber was highly important in determining whether dietary fiber was considered during food choice. Relative to an individual who acknowledged zero FDA-recognized benefits, individuals who recognized 1 to 6 benefits were between 3 and 14 times more likely to report considering dietary fiber when making food choices (Figure 3). In fact, for each additional FDA-recognized benefit identified, odds ratios for the consideration of fiber during food choice increased by an average of 2.31 (ranging from 1.19 to 3.72 per additional health benefit).

By comparison, demographic variables were also significantly associated with consideration of fiber during food choice, but the magnitude of the effects were far smaller than the number of benefits of fiber believed (Figure 3). Female respondents were 1.17 times more likely to consider dietary fiber when making food choices than respondents who were not females (95% confidence interval (CI) = (1.11, 1.24)). Only the oldest category of respondents—those 65 years of age or older—were significantly more likely to consider dietary fiber during choice than the omitted category (19–24 years), with an odds ratio of 1.40 (95% CI = 1.09, 1.83).

Given the widespread media attention to the microbiome and the extensive recognition of the microbiome as a potential health benefit of dietary fiber, we repeated the analysis of the relationship between number of fiber health benefits—with the addition of the “supporting healthy gut bacteria” to the six FDA-recognized benefits—and consideration of dietary fiber during food choice. The results suggest that the belief that dietary fiber supports healthy gut bacteria strongly motivates consideration of fiber during choice, increasing the range of odds ratios for perceiving 1–7 benefits to 3.90 (for 1 benefit) to 24.32 (7 benefits). We report the odds ratios and 95% CI for both regressions in Table S1.

## 4. Discussion

The results support our hypotheses that many people are unaware of the multiple health benefits of dietary fiber and that awareness of health benefits is an important motivator for considering fiber while making food choices. While a majority of respondents believed improving bowel movements was a benefit of dietary fiber, each of the other five FDA-recognized health benefits was identified by fewer than 40% of respondents in the population-weighted IRi survey. Moreover, the most widely recognized benefit—promoting bowel movements—may be perceived as less important, particularly for individuals who do not regularly experience constipation, and therefore not a motivating factor for increasing fiber intake. However, it is important to note that belief in any health benefit markedly increased the likelihood of considering dietary fiber during food choice.

Our findings, combined with previous research reporting positive correlations between nutrition knowledge and healthy diets, suggest that limited knowledge about the health benefits of fiber may limit the amount of fiber in people’s diets [15]. In fact, our results suggest that identification of the health benefits of dietary fiber motivates purposeful consideration of fiber during food choice. Although our results are from US primary shoppers, a lack of understanding about the many health benefits of dietary fiber may be one reason for the chronically low intake among the US population [13]. Greater knowledge and belief in the other benefits of fiber, such as reducing the risk of heart disease, stroke, diabetes, and obesity [1,8,9], may help provide sufficient motivation for some consumers to increase fiber intake.

Interestingly, while not recognized by the FDA, a majority of respondents identified promoting healthy gut bacteria as a benefit of dietary fiber. The influence of dietary fiber on the gut microbiome is not only an active area of research, but also a popular topic in mainstream books and websites [19,20]. Our results indicate that messaging used to promote dietary fiber as important for gut health has successfully reached the consumer public. Perhaps strategies that have been used to promote this benefit of fiber can be applied to the lesser-known FDA-recognized benefits to reach a broader audience.

Reducing energy intake was the least recognized benefit of dietary fiber—even less so than improving skin complexion, which has no known relationship to fiber intake. We speculate that this was a misunderstood benefit and if the wording were changed, for example to “promotes satiety,” more respondents would have identified this benefit, though given low levels of awareness of most health benefits, it would still likely be under-recognized. Future research could examine the best terminology to tailor messages that will meet the needs of consumers in general and for specific demographics [34].

Another important outcome of the analysis is the disparity in beliefs in the benefits of fiber by age group, with young adults selecting significantly fewer health benefits of dietary fiber than older adults. This is perhaps not surprising because many of the health benefits of fiber, such as reduced cholesterol and blood glucose, are long-term benefits that become more relevant as one ages [35]. Previous research has shown that messaging focused on long-term nutritional benefits is perceived as irrelevant to younger adults [36]. Additionally, younger adults patronize fast-food restaurants, which typically offer foods that are low in dietary fiber and high in energy, more often than older adults, and individuals that eat at fast-food restaurants tend to ignore nutritional information [37]. The infrequently identified benefit of dietary fiber—reducing energy intake—is one short-term benefit that may resound with a younger audience if effectively promoted. A lack of understanding of the importance of fiber by young adults may be concerning given the increase in colon and rectal cancers among younger adults in recent years [38]. Higher consumption of dietary fiber has been shown to reduce the risks of colorectal cancer [39].

Although being older or female was associated with greater likelihood of consideration of fiber during food choice compared with being younger or not female, our results clearly show that consideration of dietary fiber during choice is predominantly explained by the number of benefits respondents believed resulted from dietary fiber consumption. The effect size of identifying any benefit was markedly larger than being in the oldest age category or being female. Therefore, identification of the positive impacts of fiber consumption on health may provide motivation for individuals to seek out high-fiber foods.

A strength of our study is our large sample size of population-weighted responses from US shoppers. However, this is also a limitation. Due to the cost of adding questions to the survey and limitations to the structure of added questions imposed by the survey firm that conducted this survey, we were unable to include detailed demographic and probing questions to determine the underlying reasons for the differences in beliefs in fiber benefits among consumers. Despite this, we show a strong relationship between the number of benefits consumers believed resulted from fiber consumption and consideration of fiber during food choice, which suggests the importance of awareness in helping consumers to make better food choices.

We were also unable to ask multi-part questions. We evaluated beliefs about the health benefits of fiber using a check-all-that-apply question format where a great majority of the response options were in fact established or actively researched benefits (6 or 7 out of 8). Previous research has shown that multi-part questions such as check-all-statements questions where consumers must answer yes or no to each item in a list rather than the check-all-that-apply setup that we used affects the number of agree responses [40,41]. If a question is framed as measuring objective knowledge (e.g., “Which of the following benefits is associated with dietary fiber…”), participants may avoid answering yes to some of the benefits, speculating about the number of correct responses. Importantly, we phrased the question as a “belief” question (i.e., “Which of the following … do you believe are health benefits that result from consuming dietary fiber”) in an effort to have respondents base answers on their own beliefs instead of trying to obtain the “correct” answer. Our results show that very few people believe in the unrelated benefit of dietary fiber—improving skin complexion—but many people also do not recognize many of the true benefits of dietary fiber.

Another limitation to our study is that we only have data on self-reported consideration of dietary fiber during food choice rather than data on individuals’ real-world food choices. Real-world choices would provide more powerful evidence of the linkages between beliefs about the health benefits of fiber and the fiber content of foods purchased. However, previous research has shown that people with greater general nutrition knowledge select healthier foods and have lower incidence of diet-related diseases [42]. Based on our current findings, we plan to examine the relationship between identification of the benefits of fiber and consumer purchasing behavior in future research.

Additionally, there may be underlying individual characteristics, such as a general health motivation, that drive both beliefs about individual nutrients, such as dietary fiber, and the choice of healthier foods [43,44]. In this case, interventions to increase knowledge might not change the behaviors of low-motivation individuals. However, previous research that randomly exposed some individuals to objective information about the health benefits of dietary fiber showed that the informed individuals (1) chose food items with significantly higher levels of dietary fiber, (2) were much more likely to examine a healthier set of items during the choice process, and (3) used fiber information during the choice process more often than those not exposed to the message [45,46], which suggests that there likely are causal effects of knowledge.

## 5. Conclusions

This study confirmed our hypothesis that many individuals are unaware of the numerous and diverse health benefits of dietary fiber. Indeed, a majority of respondents identified no benefits or only one FDA-recognized benefit of dietary fiber. This study also revealed that belief in the health benefits is critical to considering healthier foods, as individuals that identified all six FDA-recognized benefits of dietary fiber were 14 times more likely to consider the fiber content of foods when making food choices than those who recognized no benefits of fiber. Although respondents across all demographic categories did not recognize many of the benefits of fiber, younger adults identified the lowest number of health benefits of fiber.

These findings suggest that education and messaging focused on the less well-known, but highly important, health benefits of dietary fiber—especially targeted at younger adults—may promote higher levels of dietary fiber consumption and ultimately improve public health. Our finding that a majority of consumers identified “supporting healthy gut bacteria” as a consumer-perceived benefit of fiber is encouraging. Although not an FDA-recognized benefit of fiber, consumers consider this a relatively new indirect benefit of fiber that has been discussed widely in popular media, including news articles and books. Thus, similar messaging used to promote healthy gut bacteria could be used to promote the lesser-known benefits of fiber.

## Figures and Tables

**Table 1:** Summary of the gender and age of participants in the IRi Survey (*n* = 42,018).

Category	Percentage (*n*)
Gender	
Female	79.9% (33,582)
Male	19.9% (8362)
Other	0.2% (74)
Age	
19–24 year	0.9% (393)
25–34 year	8.6% (3601)
35–44 year	16.1% (6777)
45–54 year	18.1% (7621)
55–64 year	25.5% (10,719)
65y and above	30.7% (12,907)

**Table 2:** Multivariate ordinal logistic regression of total FDA-recognized benefits of dietary fiber on demographic characteristics in the IRi survey (*n* = 42,018).

Category	Prop. OR (95% CI) ^a^
FEMALE (Ref.: Not female)	1.178 (1.128, 1.230)
AGE (Ref.: 19–24 years)	
25–34 years	1.429 (1.183, 1.728)
35–44 years	1.668 (1.387, 2.008)
45–54 years	2.173 (1.808, 2.614)
55–64 years	2.508 (2.089, 3.014)
65 years and above	2.405 (2.005, 2.890)

^a^ Proportional odds ratio (95% confidence interval).

## Data Availability

The data presented in this study are available on request from the corresponding author. The data are not publicly available due to contractual restrictions.

## Supplementary Materials

The following supporting information can be downloaded at: https://www.mdpi.com/article/10.3390/nu14112341/s1, Table S1: Multivariate binary logistic regression of consideration of dietary fiber when making food choices (versus not considering fiber) in the IRi survey using only FDA-recognized benefits and FDA-recognized benefits + that of supporting healthy gut bacteria (microbiome).

## Appendix A. Questions Asked to Participants in the IRi Survey

1. Which of the following, if any, do you believe are health benefits that result from consuming dietary fiber? (Select all that apply)
  - Lowering blood glucose
  - Lowering cholesterol levels
  - Lowering blood pressure
  - Increasing frequency of bowel movements
  - Increasing mineral absorption in the intestinal tract
  - Reducing energy intake
  - Maintaining healthy gut bacteria
  - Improving skin complexion

[answers randomized]

1. Which of the following nutrients, if any, do you consider when you are choosing what foods to buy or eat (or have you considered in the past when establishing dietary patterns that you currently follow)? (Select all that apply)
  - Potassium
  - Calcium
  - Dietary fiber
  - Vitamin D
  - Calories
  - Saturated fat
  - Sodium
  - Added sugar

[answers randomized]
