# Supplementary material for: US Consumer Identification of the Health Benefits of Dietary Fiber and Consideration of Fiber When Making Food Choices
Source: Nutrients. 2022 Jun 3;14(11):2341. doi: 10.3390/nu14112341 (PMC9182951; doi:10.3390/nu14112341)
Supplement: Supplementary file 1 [file nutrients-14-02341-s001.zip › nutrients-1743081-Supplementary Materials.pdf]

nutrients

MDPI

Supplementary Materials

US Consumer Identification of the Health Benefits of Dietary Fiber and Consideration of Fiber When Making Food Choices

Christopher R. Gustafson [1,*] and Devin J. Rose [2,3,4,*]

Table S1. Multivariate binary logistic regression of consideration of dietary fiber when making food choices (versus not considering fiber) using only FDA-recognized benefits and FDA-recognized benefits + supporting healthy gut bacteria (microbiome).

Category	FDA-recognized OR (95% CI)[a]	FDA-recognized+Microbiome OR (95% CI)
**BENEFITS RECOGNIZED (Ref: No benefits)[b]**		
One Benefit	3.01 (2.77, 3.28)	3.90 (3.47, 4.40)
Two Benefits	5.02 (4.61, 5.47)	6.55 (5.85, 7.35)
Three Benefits	7.14 (6.53, 7.82)	10.03 (8.95, 11.27)
Four Benefits	9.42 (8.53, 10.41)	13.79 (12.24, 15.58)
Five Benefits	13.14 (11.69, 14.79)	17.18 (15.12, 19.57)
Six Benefits	14.34 (12.39, 16.61)	23.72 (20.59, 27.39)
Seven Benefits	NA[c]	24.32 (20.63, 28.72)
**FEMALE (Ref.: Not female)**	1.17 (1.11, 1.24)	1.12 (1.05, 1.18)
**AGE (Ref:19–24 y)**		
25–34 y	1.10 (0.85, 1.45)	1.09 (0.83, 1.43)
35–44 y	1.02 (0.79, 1.34)	1.00 (0.77, 1.31)
45–54 y	1.03 (0.79, 1.34)	1.00 (0.77, 1.31)
55–64 y	1.15 (0.89, 1.5)	1.12 (0.86, 1.46)
65 y and above	1.40 (1.09, 1.83)	1.35 (1.04, 1.76)

[a]Odds ratio (95% confidence interval); [b]Reference category; [c]Not applicable.
